# Simultaneously ultrasensitive and differential detection of SARS-CoV-2, adenovirus and influenza a virus using multiplex fluorescence lateral flow immunoassay

**DOI:** 10.3389/fimmu.2025.1540676

**Published:** 2025-05-09

**Authors:** Xiaoyan Li, Changxu Zhao, Guangzheng Hou, Zhihua Sun, Xiaomei Liu, Yanlei Ding, Yi Fang, Qiqi Liu

**Affiliations:** ^1^ Bioinformatics Center of Academy of Military Medical Sciences, Beijing, China; ^2^ Beijing Key Laboratory of New Molecular Diagnosis Technologies for Infectious Diseases, Beijing, China; ^3^ Infectious Diseases Department of Air Force Medical Center, Beijing, China

**Keywords:** lateral flow immunoassay, quantum dot nanobeads, respiratory viruses, SARS-CoV-2, differential detection

## Abstract

**Introduction:**

The rapid and precise differential diagnosis of respiratory diseases is crucial to impede the spread of the viruses, considering the substantial demand resulting from frequent co-infections

**Method:**

A quantum dot nanobeads (QBs)-based multiplex fluorescence lateral flow immunoassay (QBs-based MF-LFA) biosensor was developed. The MF-LFA biosensor enabled simultaneous and sensitive quantification of severe acute respiratory syndrome coronavirus 2 (SARS-CoV-2), Adenovirus (ADV), and Influenza A Virus (IAV), boasting low limit of detection (LOD) of 56, 120, and 41 copies/mL, respectively. Compared to colloidal gold LFA, the LOD was improved by 200, 417, and 1220 times, respectively, while maintaining sensitivity comparable to PCR techniques.

**Result and discussions:**

The biosensor provided results within 20 minutes, exhibited good reproducibility, and boasted high accuracy with recoveries ranging from 96% to 105%. Additionally, the biosensor had a shelf life of up to 8 months, attributed to the use of freeze-dried probes with minimal water content, ensuring enhanced stability. Clinical samples of SARS-CoV-2, ADV and IAV infections were tested, the results were consistent with both PCR testing and clinical diagnostic tests. This highlights the considerable potential of our biosensor for early and rapid differential detection of respiratory viruses.

## 1 Introduction

Respiratory viruses are highly contagious, with a short incubation period and rapid onset of symptoms. They primarily spread through respiratory droplets and bodily fluids, causing approximately 4 million deaths annually (1). The global outbreak of Coronavirus Disease 2019 (COVID-19) has once again raised comprehensive concerns about pathogenic respiratory viruses. Severe Acute Respiratory Syndrome Coronavirus 2 (SARS-CoV-2) is the culprit behind COVID-19. New variants of SARS-CoV-2 are still emerging (2), which may lead to antigenic escape and a reduction in the sensitivity of detection methods, thereby increasing the complexity of clinical diagnosis (3). Moreover, SARS-CoV-2 can co-infect with other respiratory viruses, with a co-infection rate of 5% (4). In a study (5) conducted in Saudi Arabia, 48 hospitalized COVID-19 patients were screened for 24 respiratory pathogens, revealing that 71% of the patients had co-infections. The study also showed that in patients co-infected with COVID-19 and influenza, the average admission rate to the intensive care unit (ICU) was 6.2%, and the mortality rate was 12.3%. Compared with non-single infections, the odds of a fatal outcome in SARS-CoV-2 positive patients who are co-infected with another respiratory virus increased by 25% (6). This indicates that co-infections may lead to more severe clinical outcomes, and misdiagnosis can further increase this risk. Given the initial symptoms shared by these respiratory viruses (7–9), such as fever, cough, headache, and sore throat, it is difficult to distinguish between single and co-infections based solely on symptoms, which can easily lead to misdiagnosis. Considering the severity of co-infections and the risk of misdiagnosis, it is crucial to develop detection methods that can simultaneously identify multiple respiratory pathogens (10). IAV is the predominant influenza infection during the flu season, and in winter respiratory virus infections, SARS-CoV-2, ADV, and IAV are commonly implicated. Influenza can be treated with prescription antivirals like oseltamivir to enhance treatment effectiveness, reduce the observation time for suspected patients, and prevent antibiotic misuse (11). The recommended antiviral treatment window is within two days of symptom onset (12). Moreover, there are also some pharmacological treatments for COVID-19 that have shown significant efficacy in reducing hospitalizations and severe outcomes (13). Therefore, rapid identification and detection of SARS-CoV-2, ADV, and IAV are crucial for disease treatment and epidemic prevention and control.

However, the current gold standard diagnostic method in hospitals, viral culture, takes several days (14). Other testing methods include Polymerase Chain Reaction (PCR) (15, 16) and Enzyme-Linked Immunosorbent Assay (ELISA) (17, 18), which offer high sensitivity but also require strict conditions, time-consuming steps, precise instruments, and skilled personnel, making them less accessible in resource-limited environments. Lateral flow immunoassays (LFAs) have emerged as a promising point-of-care testing (POCT) method widely used in clinical diagnostics, personal health monitoring (19–21). There is a strong demand for a sensitive and accurate LFA biosensor capable of differentiating common respiratory viruses, especially in the face of COVID-19 pandemic. Nevertheless, conventional LFA test strips offer only semi-quantitative analysis with a high limit of detection (LOD) (22–24), limiting their sensitivity and quantification. Moreover, most LFAs currently available are primarily designed for the detection of single pathogens, which presents a significant technological gap when dealing with mixed infections involving multiple pathogens (25). For instance, LFAs targeting the antigen of SARS-CoV-2 or the influenza virus are common (26). This single-target detection approach is prone to misdiagnosis or missed diagnosis when confronted with mixed infections. Building upon this, the present study developed a quantum dot nanobeads (QBs)-based multiplex fluorescence lateral flow immunoassay (QBs-based MF-LFA) biosensor for simultaneous detection of SARS-CoV-2, ADV, and IAV. This addresses the drawbacks of lengthy RT-PCR diagnostics and the low sensitivity, as well as the limitation of traditional LFAs that can only detect a single pathogen.

The 2023 Nobel Prize in Chemistry has been awarded to scientists for their outstanding contributions to the discovery and synthesis of quantum dots. Semiconductor quantum dots with diameters ranging from 1 to 20 nm exhibit several significant features, including high quantum yield, broad excitation, and narrow, adjustable fluorescence emission spectra. These features make them suitable as a marker for LFA to achieve high sensitivity and quantitative detection (27, 28). However, quantum dots face challenges, like complex purification procedures and limited stability in biological samples (29). To overcome these limitations, researchers have harnessed QBs, which are polymer or silica nanobeads embedded with hundreds of quantum dots. QBs have been employed as a stable and highly luminescent maker in LFA to improve the accuracy and sensitivity of quantitative analysis. Some studies have demonstrated that the QBs-based LFA biosensor can enhance sensitivity by 100 times in the detection of viruses compared to a traditional colloidal gold LFA (30–32).

In general, LFA strip often involves the coating of antibody probes onto a binding pad (33, 34). The test sample undergoes chromatography and reacts with the probes on the binding pad, resulting in a short reaction time. However, this can lead to incomplete immune reactions, resulting in lower detection sensitivity and a higher likelihood of false negatives, especially for samples with low concentrations. In recent years, some studies (29, 35, 36) have taken a different approach by keeping the probes separate from the strip and thoroughly mixing them with the sample before detection. This significantly enhances detection sensitivity. However, since the probes are in liquid form, they require strict storage conditions at 4 °C which is inconvenient for product transportation and limits the range of application scenarios. Additionally, the product shelf life is not extended. To address the issues, this study prepared the freeze-dried probes. The QBs-based MF-LFA biosensor developed in this study consisted of a MF-LFA strip (with three test lines and one control line) and a tube of independent freeze-dried probes. It’s worth noting that the freeze-dried probes enabled pre-incubation of the sample, allowing for the detection of ultra-low virus concentrations after completed antigen-antibody reactions. The proposed strip successfully achieved the differential and quantitative detection of frequent co-infections involving SARS-CoV-2, ADV, and IAV. Through optimization, the sensitivity of the biosensor was essentially equivalent to the PCR detection, demonstrating the significant potential for accurately diagnosing respiratory diseases in POCT scenarios.

## 2 Materials and methods

### 2.1 Materials, reagents and instruments

SARS-CoV-2 capture monoclonal antibody (Catalog #MR900604), detection monoclonal antibody (Catalog #MR900602) and SARS-CoV-2 Nucleoprotein (NP) antigen (Catalog #MR900102) were purchased from Maiyue Biological Co. ADV capture monoclonal antibody (Catalog #A477), detection monoclonal antibody (Catalog #A016) and goat anti-mouse IgG (Catalog #1713) were obtained from Xinxin Bio, Co. IAV capture monoclonal antibody (Catalog #G1-007) and detection monoclonal antibody (Catalog #G1-008) were purchased from Feipeng Biological Co. Water-soluble carboxylated CdSe/ZnS QBs (FM610C, 365 nm excitation and 610 nm emission) were bought from Beijing Najing Biotechnology Co. The nitrocellulose (NC) membranes (CN95) was obtained from Sartorius, Germany, and the sample pad, absorbent pad, and plastic backing plate were purchased from Shanghai Jieyi Biotechnology Co. N-hydroxysuccinimide (NHS) and 1-ethyl-(3-dimethylaminopropyl) carbodiimide hydrochloride (EDC) were purchased from Beijing Bailingway Technology Co. Dimethylsulfoxide (DMSO) was provided by Beijing Inokai Technology Co. Morpholine Ethanesulfonic Acid (MES) was purchased from Shanghai Aladdin Biochemical Technology Co. Polyethylene glycol-20000 (PEG-20000), sucrose, and bovine serum albumin (BSA) were obtained from Sinopharm Chemical Reagent Co. Tween-20 (Tween-20) was purchased from Sigma, USA. ProClean 950 (PC-950) and 10% 1,2-Benzisothiazolin-3-one (BIT-10) preservatives were provided by Suzhou Genemill Biotechnology Co., Ltd.

Transmission electron microscopy (TEM) images of the nanomaterials were taken on Tecnai G2 F20 microscope (Philips, Holland). Zeta potential and dynamic light scattering (DLS) data were investigated using Mastersizer 2000 (Malvern, UK). The fluorescence signals of strips were simultaneously recorded by using a portable FIC-S1 fluorescence reader, which were purchased from Suzhou Hemai Precision Instrument Co., Ltd (China). The fluorescence images were captured by the fully automatic gel imaging system (Fusion FX Spectra) which is produced by VILBER LOURMAT (French). The respiratory viruses were quantified by the micro-drop digital PCR (dd-PCR) platform (model: TD-1) purchased from Beijing Xinyi Technology Biotechnology Co., (China). An ABI-7500 real-time fluorescent quantitative PCR (RT-qPCR) instrument was provided by Applied Biosystem Inc. (America) used to obtain the CT value of samples. The freeze-dryer (model: LGJ-40G) purchased from Foring Technology Development (Beijing) Co., Ltd (China) was used to prepare freeze-dried probes. The fluorescence spectrometer (model: SR-4VN500-10) purchased from Weihai Optical Instrument (Shanghai) Co., Ltd. was used to detect QBs and probes.

### 2.2 Preparation of QBs-Ab probes

The monoclonal antibodies of the three target respiratory viruses were individually conjugated with QBs via carbodiimide chemistry to prepare QBs-SARS-CoV-2, QBs-IAV, QBs-ADV conjugates. In brief, for each type of antibodies, 25 µL MES (20 mmol/L, pH 6.0) and 25 µL QBs (1 µmol/L), ultrasonically mixed for 3min. Then 1µL of EDC (20 mg/mL) and 1μL of NHS (20 mg/mL) were added and activated at 37°C for 15 minutes. Then, 10 μg antibody was added to the supernatant, and the mixture was incubated for 2 h at 37 °C under shaking at 800 rpm. The unreacted carboxyl groups on the QBs surface were blocked with 10% BSA for another 30 minutes. Finally, the conjugates were washed two times with borate buffer (5 mmol/L, pH 8.0). The prepared three conjugates were mixed and resuspended with 25 μL of storage solution (borate buffer containing 1% BSA) as QBs-Ab probes for follow-up test.

### 2.3 Preparation of freeze-dried probes

The prepared probes were diluted 2000-fold using a running buffer (TrisHCl) containing sucrose, trehalose, Tween-20, BSA, and PEG-20000. To ensure stability, 1 vt% PC-950 and 1 vt % BIT-10 were introduced as preservatives. The freeze-dried protective agent composition consisted of a phosphate buffer solution (0.01 mol/L) containing 5 wt% sucrose, 4 wt% mannitol, and 8 wt% proline, which were added to the mixture in a 50% volume ratio. The solution was then dispensed at 60 μL per tube for packaging before undergoing the freeze-drying process to produce the final freeze-dried probes.

### 2.4 Preparation of the MF-LFA strip

**Scheme 1:**
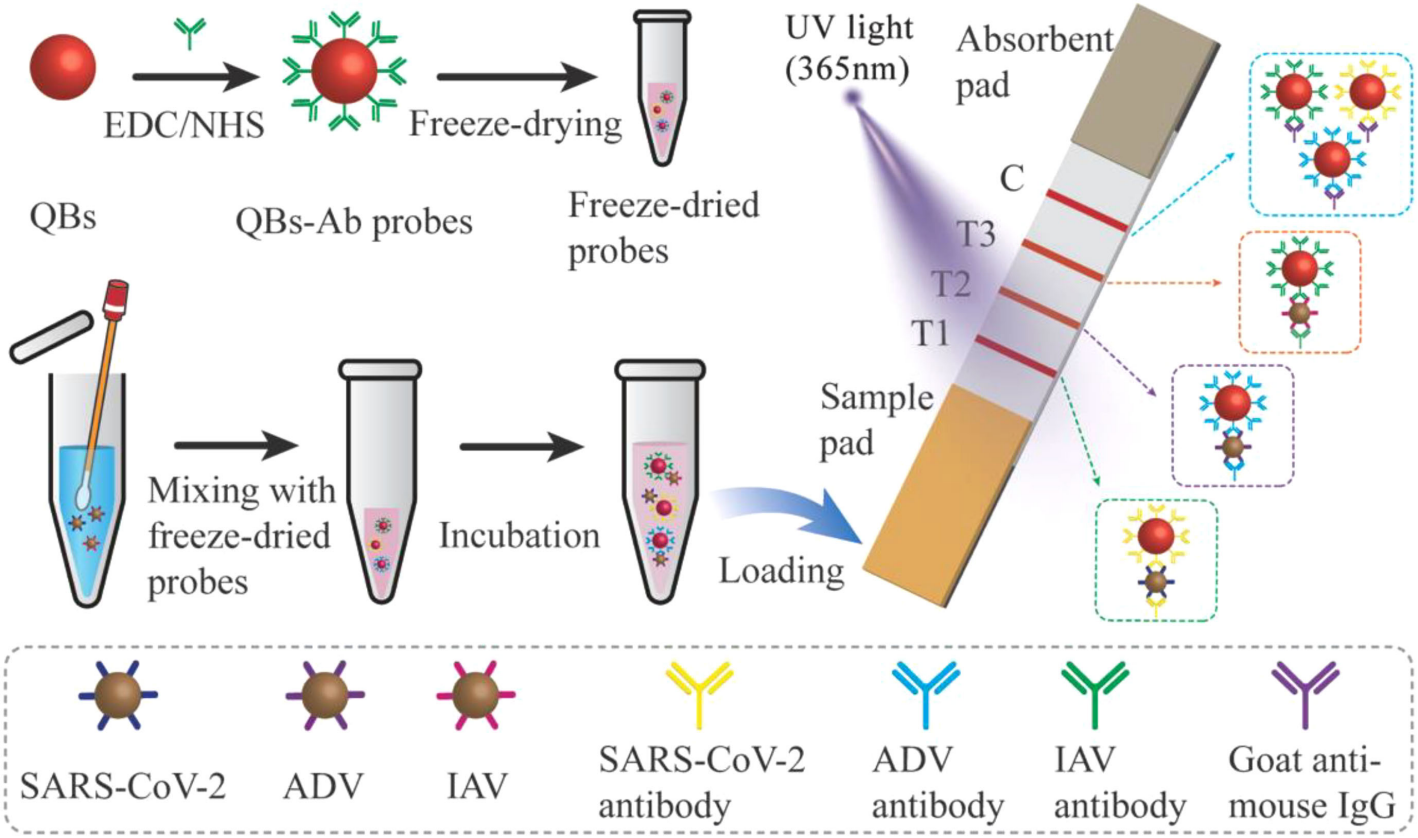
Schematic illustration of the QBs-based MF- LFA biosensor for simultaneous detection of SARS-CoV-2, ADV and IAV by using freeze-dried probes.

The MF-LFA strip was composed of a sample pad, an NC membrane with three test lines (T lines) and a control line (C line), and an absorbent pad (**Scheme 1**). The three T lines and one C line on the NC membrane were coated separately using sprays of 0.5 mg/mL SARS-CoV-2, 0.8 mg/mL ADV, 0.8 mg/mL IAV capture antibodies, and 0.2 mg/mL polyclonal goat anti-mouse IgG. Dispensed at 0.1 μL/mm rate, each antibody was applied to the NC membrane. Post-coating, the NC membrane was dried at 37°C for 2 hours and stored. Later, the sample pad, NC membrane, and absorbent pad were assembled onto a plastic backing card and sectioned into individual strips measuring 3 mm in width and 8 cm in length for future use.

### 2.5 Analytical procedure for simultaneous detection of three respiratory viruses

The concentrations of SARS-CoV-2, ADV, and IAV were measured using a dd-PCR instrument. As shown in **Supplementary Material** Section S1.1 and **Supplementary Figure S1a-c**, the three virus samples had initial concentrations of 8.0 × 10^6^, 5.0 × 10^9^, and 1.0 × 10^7^ copies/mL, respectively. Additionally, the SARS-CoV-2 NP antigen was present at a concentration of 1 mg/mL. These virus samples and SARS-CoV-2 NP antigen were diluted across various concentration gradients: 0 to 10^6^ copies/mL for viruses and 0 to 100 ng/mL for antigen, using a phosphate buffer (PB) solution (10 mmol/L).

Subsequently, 60 μL of virus samples were combined with the prepared freeze-dried probes, incubating for 5 minutes at 800 rpm to create a uniform solution through mixing. This solution was then applied to the sample pad, initiating a 15-minute chromatographic reaction. The fluorescence signals from the three test lines were captured using a commercial fluorescent signal reader, employing 365 nm excitation.

### 2.6 Detection of real biological samples and clinical samples

Different concentrations of SARS-CoV-2, ADV and IAV virus samples were added into real nasal swab samples from heathy volunteers. The mixtures were added into freeze-dried probes, then tested by the established MF-LFA strip. The recovery of three target respiratory viruses in nasal swab samples was calculated from the established calibration curves.

We also used the prepared MF-LFA biosensor for testing on clinical samples. Additionally, a PCR assay was employed to assess the consistency between the results obtained from the biosensor and PCR. All sample procedures were obtained from Huludao Central Hospital and conducted following the principles of the Declaration of Helsinki and received approval from the Ethics Committee of the Huludao Central Hospital (approval ID: LW2023-33).

### 2.7 Signal acquisition and statistical analysis

Following a 15-minute chromatographic reaction, the test strip was placed into a commercial fluorescent signal reader. The fluorescence readings of the C line and T lines were acquired and recorded. Subsequently, the test strip was promptly photographed using a gel imager. Each data set underwent three parallel experiments. The experimental data obtained was processed and visualized using GraphPad Prism (version: 9.1.1). Image editing was conducted using Adobe Photoshop (version: 2017.1.0), while schematic drawings were created using Adobe Illustrator (version: 2019).

## 3 Results

### 3.1 Principle of QBs-Based MF-LFA biosensor

We introduced fluorescent QBs as nanoprobes in the LFA platform. These nanoprobes offered distinct advantages, including high fluorescence intensity, stability, ease of antibody binding, and good dispersion. The abundance of antibodies on the QBs surface enhanced the efficiency and specificity of antigen capture (31). We transformed the probes into a freeze-dried format. The freeze-dried probes facilitated the incubation of target antigens and probes before testing and are easy to store and transport.

The operating principle of our MF-LFA biosensor for the simultaneous differential detection of SARS-CoV-2, ADV and IAV is schematically illustrated in **Scheme 1**. Following the washing of the nasal swab with physiological saline, the eluent was introduced to dissolve the freeze-dried probes. Subsequently, an incubation period was implemented, and the resulting mixture was then added dropwise to the sample pad for testing. If the target virus was present in the sample, the probes would bind to the virus antigen to form an antibody-antigen complex. As it migrated to the corresponding T line, the antigen was captured by its capture antibody, forming an antibody-antigen-antibody complex that generated a visible signal under ultraviolet light. Excess probes continued migrating to the C line, where they were captured by goat anti-mouse IgG. If the sample lacked the target antigen, the probes passed through the T line, then were captured by the goat anti-mouse IgG at the C line, again producing a signal under ultraviolet light. The fluorescence signal intensities of the three T lines could be simultaneously detected and recorded using a commercial fluorescent signal reader for quantitative viral analysis.

### 3.2 Characterization of QBs and the QBs-Ab probes

**Figure 1:**
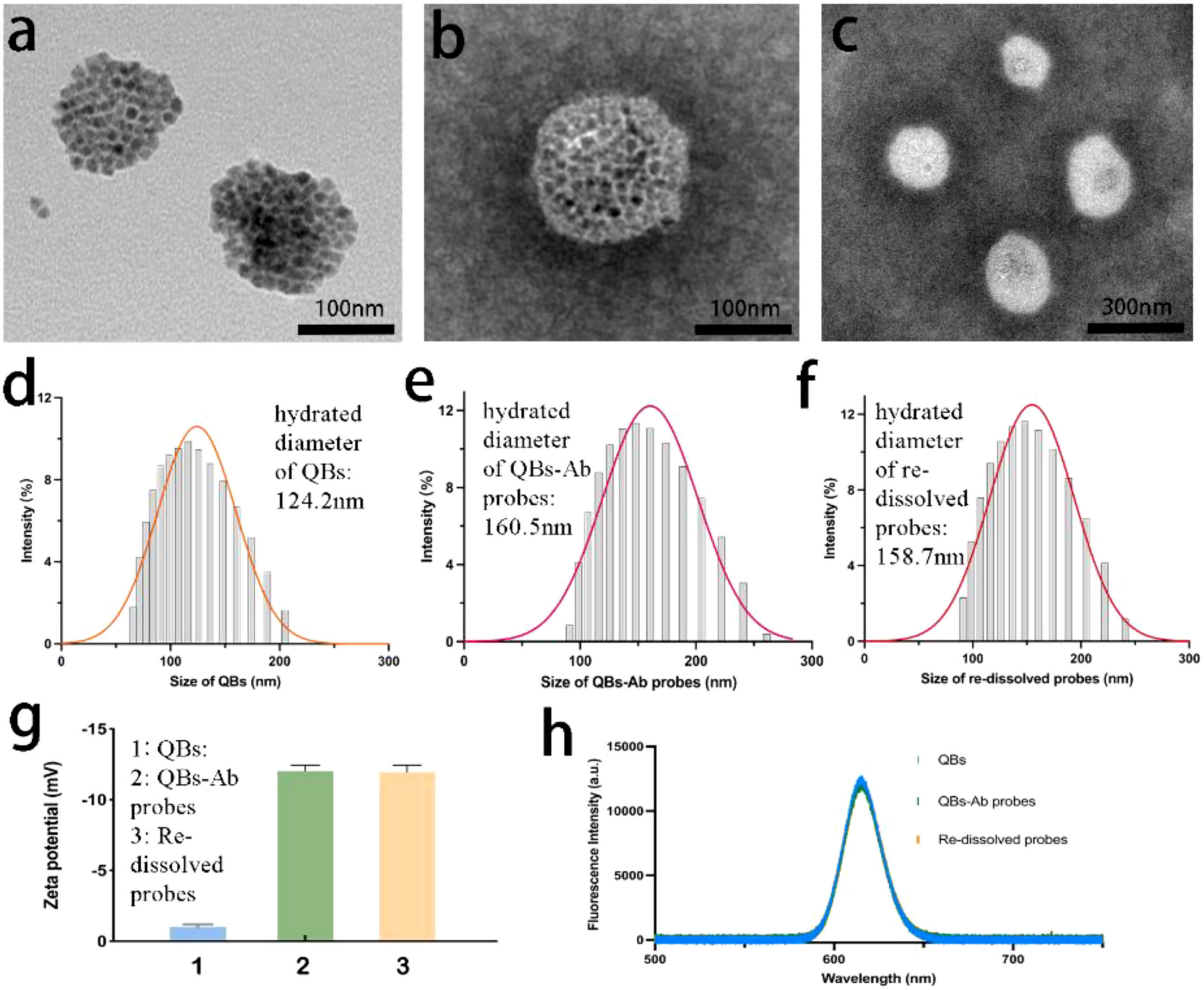
Structural characterization of QBs and the prepared QBs-Ab probes. TEM images of **(a)** QBs, **(b, c)** QBs-Ab probes. Hydrated diameter of **(d)** QBs, **(e)** QBs-Ab probes and **(f)** re-dissolved probes. **(g)** Zeta potential of QBs and QBs-Ab probes and re-dissolved probes. **(h)** Fluorescence spectra of QBs, QBs-Ab probes and re-dissolved probes.

Morphological characterization of both QBs and probes was performed using a transmission electron microscopy. As shown in **Figure 1a**, the TEM image illustrates QBs, while **Figures 1b, c** provide different magnifications of the probes. Significantly, TEM images emphasized distinct differences between the two. The QBs-Ab probes demonstrated a rougher surface texture and larger diameter, providing compelling evidence for the successful antibody conjugation to QBs.

Moreover, we utilized a particle size analyzer to examine the size distribution and zeta potential of these nanoparticles. Analysis results disclosed that QBs had an average hydrated diameter of 124.2 nm, while the probes and re-dissolved probes displayed a diameter of 160.5 nm and 158.7 nm (as depicted in **Figures 1d-f**, respectively). Notably, there was a distinct zeta potential change post-conjugation, transitioning from -1.03 mV to -12.03 mV for probes and -11.97 mV for re-dissolved probes (illustrated in **Figure 1g**). Collectively, this dataset convincingly supports the efficient and successful completion of the conjugation reaction between QBs and antibodies. **Figure 1h** displays the fluorescence spectra of QBs, probes and re-dissolved probes. As expected, the fluorescent emission peaked at 610 nm, and the fluorescence signal intensity of the probes remained substantially consistent with that of the QBs. We have also characterized the QBs-SARS-CoV-2, QBs-ADV, and QBs-IAV conjugates, which can be found in **Supplementary Material** Section S1.3 and **Supplementary Figure S2**. The performance of each individual conjugate is consistent with that of the mixed probe, the QBs-Ab probes. Meanwhile, the characterization results of the re-dissolved probes and the probes before freeze-drying showed little difference. These results directly corroborate the practical efficacy and stability of the lyophilized probes.

### 3.3 Construction of MF-LFA Biosensor for multiplex detection of respiratory viruses

**Figure 2:**
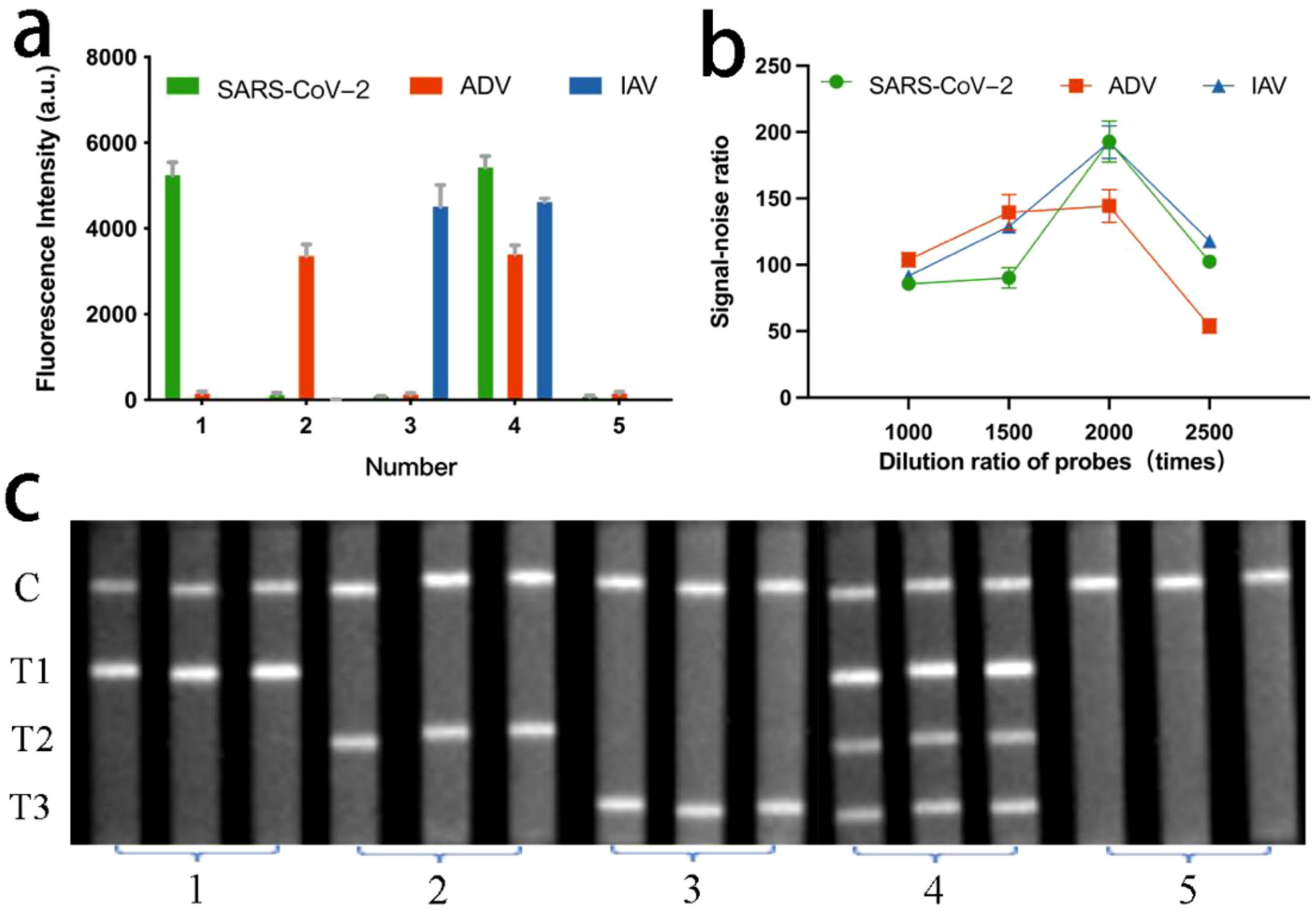
**(a)** The florescence intensities and **(c)** corresponding images of LFA strip for evaluating the cross-reactivity of three target respiratory viruses. **(b)** Signal-noise ratio of LFA strip at different dilution ratio of the probes.

**Scheme 1** describes the principle of a typical sandwich immunoassay for simultaneously detecting three respiratory viruses using the QBs-based MF-LFA Biosensor. Within the biosensor, two components were integral: a tube of freeze-dried probes and a LFA strip. The test strip was assembled with a sample pad, a NC membrane featuring three test lines and one control line, and an absorbent pad, all assembled on a plastic backing. This configuration enabled simultaneous detection of three respiratory viruses. The antigen-captured antibodies of SARS-CoV-2, ADV, and IAV were immobilized on the NC membrane to form the three test lines (T1 for SARS-CoV-2, T2 for ADV, and T3 for IAV). The detection effect of these three test lines on the multiplex test strip was evaluated using test samples containing SARS-CoV-2 (2.0 × 10^4^ copies/mL), ADV (5.0 × 10^5^ copies/mL), and IAV (2.5 × 10^4^ copies/mL). **Figure 2c** displays fluorescence images of the LFA strip. Upon adding samples containing the target viruses, corresponding-colored bands appeared on the test lines of the strip. Negative samples did not produce any colored bands on the test lines. Fluorescence intensities are depicted in **Figure 2a**. The detection outcomes indicate that the antibodies we employed exhibit high specificity, as there were no cross-reactions observed across the three test lines.

Traditional LFA test strips involve immobilizing probes onto a conjugate pad, followed by sample application-essentially, the sample and probes remain separate. This study, we used the freeze-dried probes, offering a pivotal advantage by facilitating sufficient incubation of the sample before loading. The composition of the loading buffer which was used to dilute the probes had a significant impact on the efficacy of the freeze-dried probes. To explore the specific components of the loading buffer, which included three virus detection concentrations (2.0 × 10^4^ copies/mL for SARS-CoV-2, 5.0 × 10^5^ copies/mL for ADV, and 2.5 × 10^4^ copies/mL for IAV), an orthogonal experiment was conducted. The factors and levels of the orthogonal experiment, the 18 sets of orthogonal experiments using SPSS software and the results produced are presented in the **Supplementary Material** Section S2.1, **Supplementary Table S4** and **Supplementary Table S5**. The final selected composition of the loading buffer included a 0.075 mol/L pH 8.5 Tris buffering system, containing 3 wt% sucrose, 1 wt% trehalose, 2 vt% Tween-20, 0.25 wt% PEG-20000, and 0.5 wt% BSA.

We also screened the probes dosage, specifically the dilution ratio of the probes. Prior to probe lyophilization, we diluted the probes at 1000-, 1500-, 2000- and 2500-times using loading buffer and mixed them with the samples for detection. From the detection results shown in **Figure 2b**, it can be observed that when the dilution ratio was 2000 times, the signal-noise ratio (the ratio of positive signal to negative signal) for all three T-lines was maximized, and the background value was sufficiently low at this point. Therefore, the optimal dilution ratio for probes was determined to be 2000 times.

We further optimized the key parameters of the MF-LFA biosensor, including the type of NC membrane, antibody coating quantity on the test lines, incubation time, and chromatographic reaction time. These optimizations were conducted to ensure both the sensitivity of the detection system and the optimal parameters for multiplex detection. A detailed description of the optimization experiments can be found in Section S2.2 and **Supplementary Figure S3** of the **Supplementary Material**. In summary, CN95 was chosen as the NC membrane type, and the antibody concentrations coated on the test lines were 0.5, 0.8, and 0.8 mg/mL for SARS-CoV-2, ADV and IAV, respectively. The incubation time was set at 5 minutes, with a chromatographic reaction time of 15 minutes. These conditions were determined to be the most suitable.

The method we proposed enables the simultaneous differentiation of three respiratory viruses. To the best of our knowledge, we are the first to report the application of freeze-dried probes in LFA platform.

### 3.4 Evaluation of the MF-LFA biosensor

**Figure 3:**
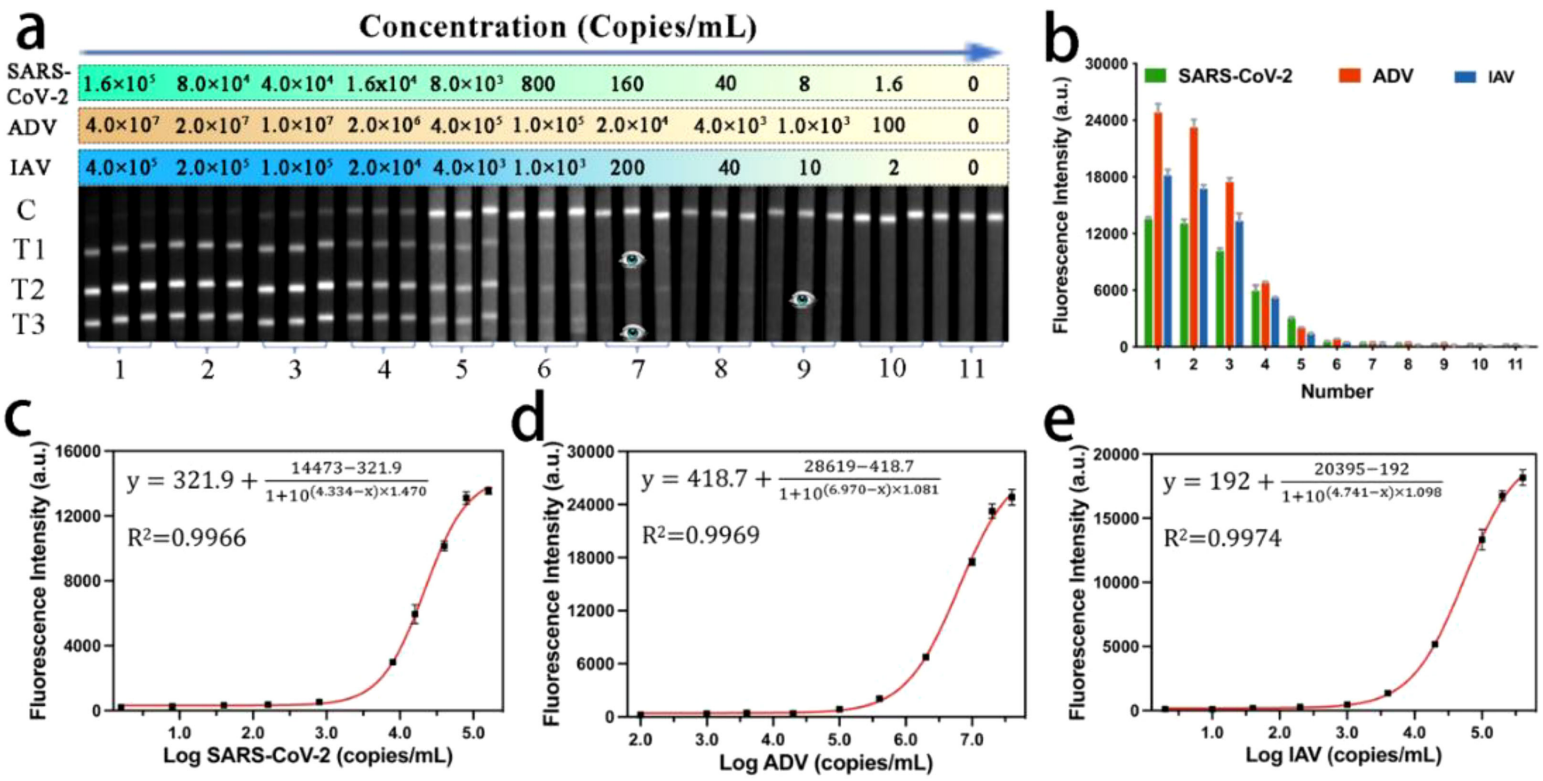
**(a)** Fluorescence images and **(b)** corresponding signal intensities on T lines of MF-LFA strips for the detection of SARS-CoV-2, ADV and IAV. **(c-e)** Calibration curves corresponding to the detection of SARS-CoV-2, ADV, and IAV.

To evaluate the detection performance of our MF-LFA biosensor for multiple respiratory viruses, we conducted tests on mixed virus samples containing SARS-CoV-2, ADV, and IAV using the proposed assay. **Figure 3** illustrates the images and the corresponding fluorescent intensities of the strips for these three target respiratory viruses. As the concentration of the mixed viruses decreased, the fluorescence intensities of the three test lines gradually diminished and eventually became undetectable. It is noteworthy that the lowest detectable viral concentration visible to the naked eye was 160 copies/mL for SARS-CoV-2, 1000 copies/mL for ADV, and 200 copies/mL for IAV, respectively. A commercial fluorescent signal reader was employed to detect and record the signal intensities of all the strips, as shown in **Figure 3b**. Subsequently, we constructed calibration curves (**Figures 3c-e**). The sigmoidal calibration curves for SARS-CoV-2, ADV, and IAV exhibited a broad dynamic range, spanning over four orders of magnitude. The correlation coefficients (R^2^) for these curves were 0.9966, 0.9969, and 0.9974, respectively. To determine the LOD of the LFA biosensor, we followed the International Union of Pure and Applied Chemistry (IUPAC) protocol (37), defining it as the concentration corresponding to three times the standard deviation of the blank groups. The calculated LODs were approximately 56 copies/mL for SARS-CoV-2, 120 copies/mL for ADV, and 41 copies/mL for IAV.

The NP antigen of SARS-CoV-2 has been demonstrated to exhibit greater conservation compared to other structural proteins, such as the spike antigen (38, 39). Several studies have indicated that NP demonstrates high sensitivity and specificity in the detection of SARS-CoV-2 (40, 41). Therefore, we also selected NP as the target protein. We employed the biosensor to detect the SARS-CoV-2 NP antigen, generating a calibration curve with a visual sensitivity of 60 pg/mL and a LOD of 5 pg/mL. (Depicted in the supplemental materials Section S3.1 and **Supplementary Figure S4**).

**Figure 4:**
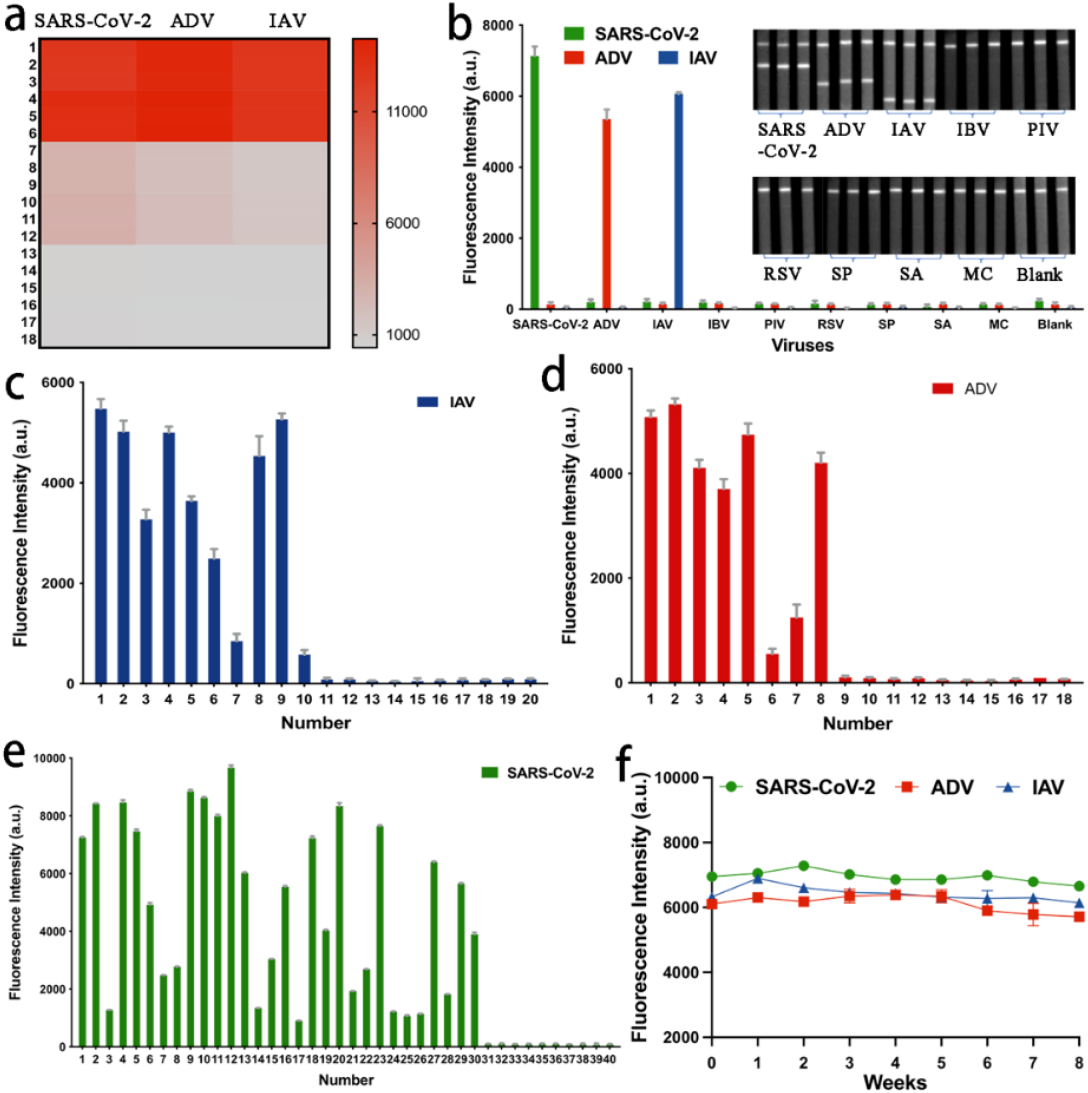
**(a)** Reproducibility of the MF-LFA biosensor for SARS-CoV-2, ADV and IAV. **(b)** Specificity of the LFA biosensor (2.0 × 10^4^ copies/mL for SARS-CoV-2, 5.0 × 10^5^ copies/mL for ADV, 2.5 × 10^4^ copies/mL for IAV and 5.0 × 10^5^ copies/mL for IBV, PIV, RSV, SP, SA, MC). Results of detection for **(c)** 20 clinical samples of IAV, **(d)** 18 clinical samples of ADV and **(e)** 40 clinical samples of SARS-CoV-2. **(f)** Stability of the LFA biosensor (2.0 × 10^4^ copies/mL for SARS-CoV-2, 5.0 × 10^5^ copies/mL for ADV, 2.5 × 10^4^ copies/mL for IAV).

We also assessed the reproducibility of the MF-LFA biosensor through six independent tests. **Figure 4a** presents the detection results of the LFA for samples with high (8.0 × 10^4^ copies/mL for SARS-CoV-2, 5.0 × 10^6^ copies/mL for ADV and 5.0 × 10^4^ copies/mL for IAV), moderate (8.0 × 10^3^ copies/mL for SARS-CoV-2, 2.0 × 10^4^ copies/mL for ADV and 4.0 × 10^3^ copies/mL for IAV), and low (8.0 × 10^2^ copies/mL for SARS-CoV-2, 1.0 × 10^3^ copies/mL for ADV and 5.0 × 10^2^ copies/mL for IAV) concentrations of mixed SARS-CoV-2, ADV, and IAV samples. The fluorescence intensities of the LFA strips within each group were consistently uniform, with relative standard deviation (RSD) values lower than 8%. We further verified the specificity of the LFA biosensor by testing other important respiratory viruses, including Influenza B virus (IBV), Parainfluenza virus (PIV), Respiratory Syncytial virus (RSV), Streptococcus pneumoniae (SP), Staphylococcus aureus (SA) and Neisseria meningitidis (MC), using the prepared strips. As depicted in **Figure 4b**, only the three corresponding viruses (2.0 × 10^4^ copies/mL for SARS-CoV-2, 5.0 × 10^5^ copies/mL for ADV and 2.5 × 10^4^ copies/mL for IAV) exhibited coloration on their respective test lines, while the results for other viruses (5.0 × 10^5^ copies/mL) were negative. Hence, the MF-LFA biosensor demonstrates excellent specificity.

Thanks to the outstanding performance of our biosensor, the developed LFA method offers significant advantages over traditional immunoassay-based POCT tools, including gold nanoparticle (AuNP)-based LFA and ELISA (42). Compared to AuNP-based LFA, our method showed an improved sensitivity of 200, 417, and 1220 times for the detection of the three viruses, respectively. When compared to ELISA, the sensitivity for detecting SARS-CoV-2 and IAV was increased by 20 and 1220 times, respectively. It is widely acknowledged that PCR is the most used method for virus detection especially since the outbreak of COVID-19, and in this study, we also utilized PCR to detect the three respiratory viruses. The limits of detection (LODs) were 40, 80, and 30 copies/mL, respectively. The PCR detection results can be found in the **Supplementary Material** Section 3.2 and **Supplementary Figure S5**. Our method’s detection sensitivity was essentially on par with that of PCR. The high sensitivity was entirely attributed to the thorough incubation of freeze-dried probes with the sample before detection.

Meanwhile, freeze-dried probes ensured thorough interaction between the target virus and the probes, consequently enhancing detection sensitivity. Moreover, the probes were prepared in a freeze-dried state, facilitating storage and extending the product’s shelf life. To verify the preservation effectiveness, the biosensor was securely sealed in an aluminum foil bag using a specialized machine, and the entire configuration including freeze-dried probes and LFA strips underwent stability testing in a controlled oven set at a temperature of 37°C. Results from stability tests indicate that the biosensor exhibited robust storage capabilities at room temperature for up to 8 months, as illustrated in **Figure 4f**, in accordance with an Arrhenius acceleration model in our previous work (43). Therefore, we proposed this freeze-dried probes-based LFA, with the aim of creating a rapid, highly sensitive, and quantitative detection method for multiple respiratory viruses.

### 3.5 Detection of real biological samples and clinical samples

To assess the practical utility of the MF-LFA biosensor with actual respiratory tract specimens, we added varying concentrations of SARS-CoV-2, ADV, and IAV into real nasal swab samples obtained from healthy volunteers. These samples were then directly tested using the established assay. Prior to testing, the nasal swab samples were subjected to RT-PCR to confirm the absence of the target viruses. By calculating the obtained fluorescence signals from each test line, the average recoveries of the proposed LFA were determined to be within the range of 96.0%-103% for SARS-CoV-2 spiked samples, 98.8%-104.6% for ADV spiked samples, and 98%-105% for IAV spiked samples, with a coefficient of variation (CV) below 7.21% (as shown in **Table 1**). These findings underscore the excellent accuracy and reliability of the biosensor for on-site detection of real clinical samples.

**Table 1:** Recovery efficiency of three respiratory viruses detected in nasal swab samples using MF-LFA biosensor.

Virus	Added concentration (copies/mL)	Detected concentration (copies/mL)	Recovery (%)	CV (%)
SARS-CoV-2	5.0 × 10^4^	4.80 × 10^4^	96.0	6.52
	5.0 × 10^3^	5.11 × 10^3^	102.2	5.25
	1.0 × 10^2^	1.05 × 10^2^	105.0	5.96
ADV	5.0 × 10^6^	4.94 × 10^6^	98.8	4.76
	5.0 × 10^4^	5.17 × 10^4^	103.4	5.52
	5.0 × 10^2^	5.23 × 10^2^	104.6	6.15
IAV	5.0 × 10^4^	4.90 × 10^4^	98.0	4.09
	5.0 × 10^3^	4.98 × 10^3^	99.6	7.21
	1.0 × 10^2^	1.05 × 10^2^	105.0	5.86

One of our primary concerns is to utilize the MF-LFA biosensor for the rapid and accurate differentiation and detection of SARS-CoV-2, ADV, and IAV in clinical samples. We conducted tests on 30 clinical nasal swab positive samples of SARS-CoV-2, 8 clinical nasal swab positive samples of ADV, 10 clinical nasal swab positive samples of IAV, and 10 clinical nasal swab negative samples of three above viruses obtained from Huludao Central Hospital. All sample procedures were conducted following the principles of the Declaration of Helsinki and received approval from the Ethics Committee of the Huludao Central Hospital (approval ID: LW2023-33). All these samples underwent RT-qPCR analysis to determine cycle threshold (Ct) values (as illustrated in **Supplementary Material** Section S1.2, **Supplementary Figure S1d-f** and **Supplementary Table S1-S3**). As depicted in **Figures 4c-e**, our biosensor testing yielded compelling results. All positive clinical samples consistently returned positive results, aligning perfectly with both PCR analysis and clinical diagnostic tests. Likewise, all negative clinical samples consistently produced negative results. This consistent alignment underscores the reliability of our MF-LFA biosensor in detecting clinical samples. Notably, even samples with relatively high Ct values, such as 37.04, 37.02, and 37 for SARS-CoV-2, ADV, and IAV, respectively, were accurately detected. This showcases the robust sensitivity of our biosensor in clinical sample testing.

The constructed MF-LFA biosensor exhibited distinct advantages, including multiplex testing, rapid detection, and high sensitivity. When compared to highly sensitive biosensors developed for respiratory virus detection in recent years (**Table 2**), our LFA biosensor clearly stands out, showcasing exceptional sensitivity, high throughput, and rapid detection capabilities. Recent PCR detection methods for respiratory viruses are also outlined in **Table 2**. In summary, our method’s sensitivity matches that of PCR, offering significantly enhanced sensitivity and stability while still allowing for multiplex detection and quantification. Furthermore, in comparison to PCR, our method offers a substantial time-saving advantage.

**Table 2:** Comparison of the performance of the proposed assay and other recently reported LFA and PCR methods for respiratory virus detection.

Detection method	Detection of targets	LODs	Assay time	References
Florescent-LFA	SARS-CoV-2 ADV IAV	8 pg/mL 488 copies/mL 471 copies/mL	15 min	(42)
Florescent-LFA	SARS-CoV-2	1 pg/mL 0.5 pg/mL	10 min 35 min	(44)
SERS-LFA	SARS-CoV-2 IAV	5.2 pfu/mL 10 pfu/mL	–	(45)
SERS-LFA	ADV IAV	10 pfu/mL 50 pfu/mL	–	(46)
Colorimetric-LFA	SARS-CoV-2	38 pg/mL	10 min	(47)
RT-PCR	ADV IAV	100 copies/mL 100 copies/mL	60 min	(48)
RT-PCR	SARS-CoV-2	100 copies/mL	30 min	(49)
RT-PCR	SARS-CoV-2 IAV	50 copies/PCR 100–200 copies/PCR	30 min	(50)
MF-LFA	SARS-CoV-2 ADV IAV	56 copies/mL, 5 pg/mL 120 copies/mL 41copies/mL	20 min	This work

## 4 Discussion

In this study, we present an ultrasensitive multiplex fluorescence lateral flow assay biosensor based on quantum dot nanobeads, capable of simultaneously detecting SARS-CoV-2, ADV, and IAV.

We prepared freeze-dried probes and incubated them with samples, ensuring thorough antigen-antibody reactions that enable the detection of ultra-low-concentration samples, thus achieving high-sensitivity detection. Our MF-LFA biosensor demonstrated LOD of 56, 120, and 41 copies/mL for SARS-CoV-2, ADV, and IAV, respectively. Compared to colloidal gold-based LFA, the LOD was enhanced by 200, 417, and 1220 times, respectively (42), which was essentially equivalent to the sensitivity of PCR assay. **Table 3** lists several commercialized LFA kits for detecting respiratory viruses, all with relatively high detection limits. Our biosensor’s LODs for SARS-CoV-2 are 56 copies/mL and 5 pg/mL. This suggests our LOD for IAV is at the pg/mL level, compared to BinaxNOW™ Influenza A&B’s ng/mL level. The BinaxNOW COVID-19 Antigen detects ≥10^5^ copies/mL of SARS-CoV-2 (57), with an LOD of 140.6 TCID_50_/mL, indicating our biosensor’s higher sensitivity. BD Veritor™ shows weaker detection in triple combined tests. VP/mL, being the ratio of copies/mL to genomic copies per viral particle, yields lower quantification values than copies/mL. Yet, our ADV LOD is significantly lower than EZER™ ADV Antigen’s, confirming our biosensor’s superior sensitivity. In summary, our biosensor maintains high sensitivity during multiplex detection.

**Table 3:** List of commercially available LFA kits.

Product	Detection of targets	LODs	Assay time	References
BinaxNOW™ Influenza A&B	IAV IBV	103 ng/ml 6.05 ng/ml	15min	(51)
BinaxNOW™ COVID-19 Antigen	SARS-CoV-2	140.6 TCID_50_/mL	15 min	(52)
BD Veritor™ System for SARS-CoV-2 & Flu A+B	SARS-CoV-2 IAV IBV	280 TCID_50_/mL 10^4^ TCID_50_/mL 10^6–^10^7^ TCID_50_/mL	15 min	(53)
BD Veritor™ System for SARS-CoV-2	SARS-CoV-2	140 TCID_50_/mL	15 min	(54)
BD Veritor™ System for Flu A+B	IAV IBV	10^2–^10^3^ TCID_50_/mL 10^3–^10^4^ TCID_50_/mL	15 min	(55)
EZER™ ADV Antigen	ADV	10^3–^10^4^ VP/mL	15min	(56)

Accelerated testing in an oven after sealing the MF-LFA biosensor showed no significant decline in detection signals even after 8 months of storage at room temperature, thanks to our freeze-dried probes, which exhibited enhanced stability due to moisture evaporation. Although, some research separates the fluorescent probe for sample incubation, these studies often neglect to investigate the stability of the product or only assess the stability of the probe under 4°C conditions (45). The inconvenience arising from the need for low temperatures makes it challenging to extend the applicability of these probes to clinical settings. Furthermore, we evaluated the clinical utility of the MF-LFA biosensor by testing samples prepared by spiking SARS-CoV-2, ADV, and IAV into nasal swabs from healthy individuals, achieving recovery ranging from 96% to 105%. The clinical detection efficacy of our method was further substantiated by evaluating a diverse set of samples, including those from 30 individuals diagnosed with COVID-19, 8 patients infected with ADV, 10 cases suffering from IAV infections, and an additional 10 clinical negative specimens. Our findings were consistent and in concordance with the results obtained through PCR, thereby reinforcing the reliability and accuracy of our testing approach. Those experimental results involving the detection of real biological samples and clinical samples also confirm the clinical utility of the biosensor, particularly in primary healthcare institutions or during the management of major infectious disease outbreaks.

However, our study has some limitations. The current study was conducted with a limited number of clinical samples, and future studies with larger sample sizes are needed to validate our findings. Additionally, the stability experiments were conducted under controlled laboratory conditions. Real-world environmental factors like humidity and light exposure may influence the performance of the biosensor and require further investigation.

Nevertheless, our proposed MF-LFA biosensor holds promise as a detection technology capable of rapidly and accurately differentiating SARS-CoV-2, ADV, and IAV using a single test strip, providing virus quantification results within 20 minutes. The sensitivity of our method is comparable to PCR. Furthermore, when tested with clinical samples, the biosensor demonstrated high stability, specificity, and accuracy. All results demonstrate the significant potential of our QBs-based MF-LFA biosensor for accurately diagnosing respiratory diseases in POCT applications.

## Data Availability

The original contributions presented in the study are included in the article/**Supplementary Material**. Further inquiries can be directed to the corresponding authors.
