# Supplementary material for: Simultaneously ultrasensitive and differential detection of SARS-CoV-2, adenovirus and influenza a virus using multiplex fluorescence lateral flow immunoassay
Source: Front Immunol. 2025 May 9;16:1540676. doi: 10.3389/fimmu.2025.1540676 (PMC12098280; doi:10.3389/fimmu.2025.1540676)
Supplement: Supplementary file 1 [file DataSheet1.docx]

Simultaneously Ultrasensitive and Differential Detection of SARS-CoV-2, Adenovirus and Influenza A Virus using Multiplex Fluorescence Lateral Flow Immunoassay

Xiaoyan Li^1,2†^, Changxu Zhao^1,2†^, Guangzheng Hou^1,2^, Zhihua Sun^3^, Xiaomei Liu^1,2^, Yanlei Ding^1,2^, Yi Fang^3^*, Qiqi Liu^1,2^*.

*^1^ Bioinformatics Center of Academy of Military Medical Sciences, Beijing, 100850, P. R. China*

*^2^ Beijing Key Laboratory of New Molecular Diagnosis Technologies for Infectious Diseases, Beijing 100850, P. R. China*

*^3^ Infectious Diseases Department of Air Force Medical Center, Beijing, 100142, P. R. China*

^†^**Co-first Authors**

Xiaoyan Li and Changxu Zhao. They contributed equally to this work and share first authorship.

^*^**Corresponding Authors**

Qiqi Liu, liuqiqi877@foxmail.com

Yi Fang, 13311168101@163.com

**Section S1 Experimental section**

**Section S1.1 Quantification of three target respiratory viruses though a micro-drop digital PCR (dd-PCR).**

Micro-drop digital PCR (dd-PCR) on a TD-1 dd-PCR platform was employed to quantify three viruses. A 30 μL dd-PCR system was prepared according to the Micro-drop Sample Preparation Universal Kit's instructions. This system was mixed with 30 μL of PCR reagents and 180 μL of microdroplet-generating oil to create water-in-oil droplets using a Drop Maker. The microdroplet samples underwent amplification in a T100 thermal cycling PCR instrument. The PCR conditions of SARS-CoV-2 and IAV were as follow: 55 °C for 30 min, 95 °C for 10 min, 95 °C for 30 s, 60 °C for 1 min (cycled 45 times), 98 °C for 10 min, and held at 4 °C. For ADV, the PCR conditions were as follow: 50 °C for 2 min, 95 °C for 10 min, 95 °C for 30 s, 60 °C for 1 min (cycled 45 times), 98 °C for 10 min, and held at 4 °C. Subsequently, signals were detected and analyzed using a Chip Reader Droplet Reader. Positive (fluorescent) and negative (nonfluorescent) droplets indicated the presence or absence of the target sequence. The number of target DNA molecules in the sample was calculated based on the fraction of positive droplets and Poisson statistics using a specific formula: λ=−ln (1− *p*). The average number of target DNA molecules per reaction (λ) and the fraction of positive reactions (*p*) were used to estimate the absolute target DNA concentration. This assessment considered the volume of each PCR reaction and the total number of replicates analyzed. The quantified result for the three virus particles were then adjusted by multiplying them by the dilution factor, resulting in initial concentrations of 8.0 × 10^6^ copies/mL for SARS-CoV-2, 5.0 × 10^9^ copies/mL for ADV, and 1.0 × 10^7^ copies/mL for IAV.

**Section S1.2 RT-qPCR detection of clinical samples**

Using Tianlong extraction reagent, nucleic acid was extracted from 40 clinical samples of COVID-19, 18 clinical samples of ADV and 20 clinical samples of IAV. Corresponding sample nucleic acids were then tested for the respective target genes using the appropriate nucleic acid test kit. Reaction mixtures were prepared with 15 μL of reaction buffer and 15 μL of sample nucleic acid. Negative and positive controls were set up. Sample testing was conducted using an ABI-7500 real-time fluorescent quantitative PCR (RT-qPCR) instrument, and CT values for all samples were obtained. The reaction conditions were as follows: 50°C for 20 minutes, 95°C for 3 minutes, 95°C for 15 seconds, 55°C for 30 seconds, cycled 45 times. RT-qPCR results are shown in **Fig. S1d-f**. The CT values obtained are presented in **Table S1-3**.


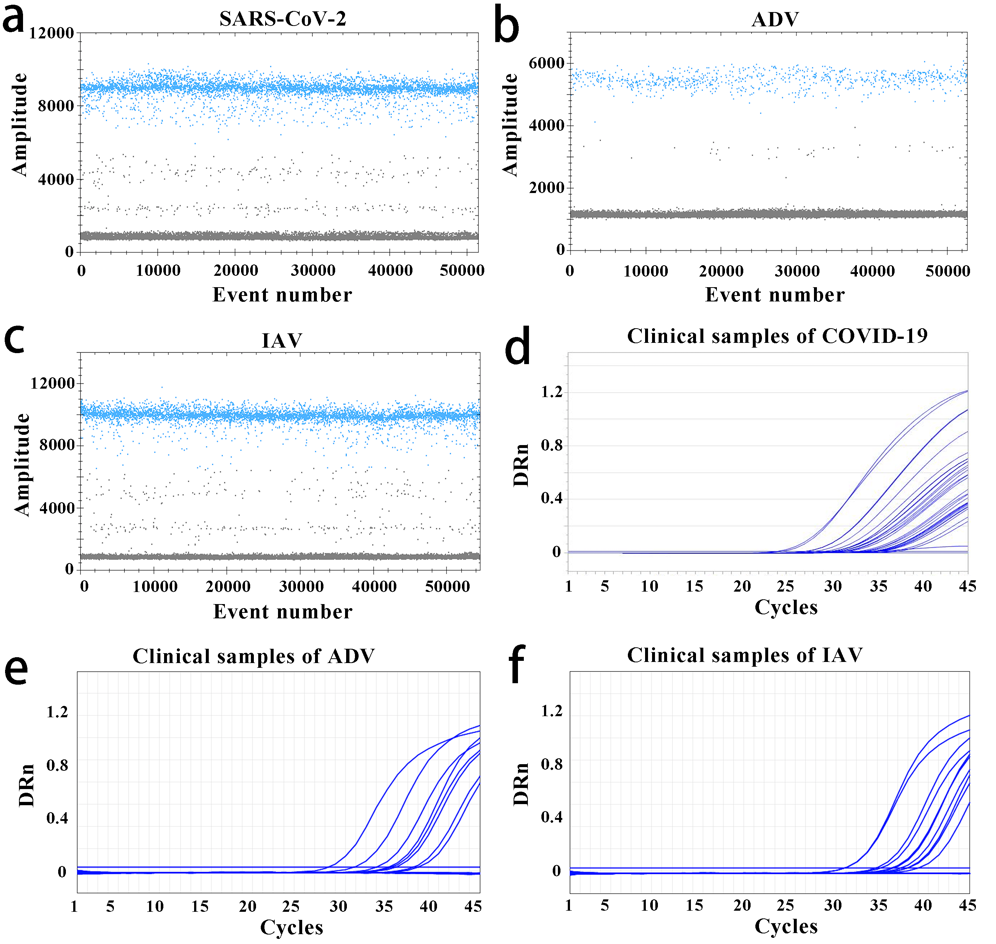


**Fig. S1.** Fluorescence signal map of the dd PCR of (a) SARS-CoV-2，(b) ADV and (c) IAV. And Amplification curves of (d) COVID-19, (e) ADV and (f) IAV clinical samples by RT-qPCR.

**Table S1** Ct values of 30 clinical samples of SARS-CoV-2

| **Number** | **CT** | **Number** | **CT** | **Number** | **CT** | **Number** | **CT** |
| --- | --- | --- | --- | --- | --- | --- | --- |
| **1** | 29.75 | **11** | 29.68 | **21** | 34.36 | **31** | NoCt |
| **2** | 28 | **12** | 23.79 | **22** | 34.29 | **32** | NoCt |
| **3** | 34.82 | **13** | 31.05 | **23** | 29.69 | **33** | NoCt |
| **4** | 27 | **14** | 34.59 | **24** | 34.94 | **34** | NoCt |
| **5** | 29.75 | **15** | 33.6 | **25** | 36.3 | **35** | NoCt |
| **6** | 32.68 | **16** | 31.54 | **26** | 35.14 | **36** | NoCt |
| **7** | 34.36 | **17** | 37.04 | **27** | 30.39 | **37** | NoCt |
| **8** | 34.29 | **18** | 30.33 | **28** | 34.54 | **38** | NoCt |
| **9** | 24.33 | **19** | 33.19 | **29** | 31.22 | **39** | NoCt |
| **10** | 26.94 | **20** | 29.05 | **30** | 33.25 | **40** | NoCt |

**Table S2** Ct values of 8 clinical samples of ADV

| **Number** | **CT** | **Number** | **CT** |
| --- | --- | --- | --- |
| **1** | 29.86 | **9** | NoCt |
| **2** | 27.38 | **10** | NoCt |
| **3** | 33.07 | **11** | NoCt |
| **4** | 34.98 | **12** | NoCt |
| **5** | 31.93 | **13** | NoCt |
| **6** | 37.02 | **14** | NoCt |
| **7** | 36.62 | **15** | NoCt |
| **8** | 32.86 | **16** | NoCt |
|  |  | **17** | NoCt |
|  |  | **18** | NoCt |

**Table S3** Ct values of 10 clinical samples of IAV

| **Number** | **CT** | **Number** | **CT** |
| --- | --- | --- | --- |
| **1** | 30.26 | **11** | NoCt |
| **2** | 32.34 | **12** | NoCt |
| **3** | 34.33 | **13** | NoCt |
| **4** | 31.78 | **14** | NoCt |
| **5** | 33.06 | **15** | NoCt |
| **6** | 35.07 | **16** | NoCt |
| **7** | 35.78 | **17** | NoCt |
| **8** | 32.56 | **18** | NoCt |
| **9** | 30.96 | **19** | NoCt |
| **10** | 37 | **20** | NoCt |

**Section S1.3 Characterization of QBs-SARS-CoV-2, QBs-ADV, QBs-IAV conjugates**

We have characterized the three antibody conjugates individually using TEM, particle size analysis, zeta potential measurements, and fluorescence spectra, with the results shown in **Fig. S2**. The TEM results show that the three conjugates have consistent morphology, with rougher surface textures and larger diameters, providing compelling evidence for the successful conjugation of antibodies to QBs. The particle sizes of the three conjugates are consistent, and their zeta potentials and fluorescence spectra are also consistent. This indicates that the conjugation effect of the three conjugates is similar, demonstrating consistency across all three conjugates.

**
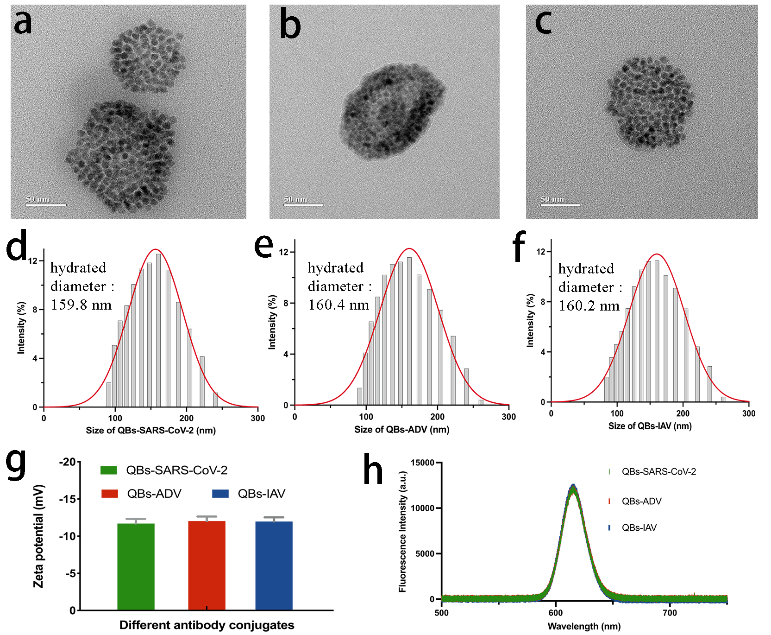
**

**Fig. S2.** Structural characterization of three conjugates. TEM images of (a) QBs-SARS-CoV-2, (b) QBs-ADV and (c) QBs-IAV. (d-f) Hydrated diameter of three conjugates. (g, h) Zeta potential and fluorescence spectra of three conjugates.

**Section S2 Optimization of the LFA biosensor**

**Section S2.1 Optimization of the loading buffer**

A 7-factor, 3-level orthogonal experiment was designed as depicted in **Table S4** to optimize the formulation of the loading buffer. Factors including pH value, ion concentration, sucrose content, trehalose content, Tween-20 content, PEG-20000 content, and BSA content were considered.

**Table S5** shows the L18 (3^7^) orthogonal experiments and the testing results. The results column represents absolute signal intensities (the total difference between positive signal intensities and negative signal intensities of the three T-lines). "ki" (i=1,2,3) represents the absolute signal intensities mean for this factor at the i-level, while "R" represents the magnitude of the absolute signal intensities mean variation for this factor at different levels.

Taking A factor in the first column as an example, we calculate "ki" and "R".

k1 = (18208+17297+18195+19043+19007+19517) / 6 = 18545

k2 = (20333+18113+19339+20548+18116+20368) / 6 = 19470

k3 = (18864+17772+18224+19828+19631+17368) / 6 = 18615

R = max (ki) – min (ki) = 19470 – 18545 = 925

Based on the magnitude of the R values, one can determine the priority order of factors influencing the experimental results. In this experiment, it was evident that the concentration of polyethylene glycol (PEG-20000) and the pH value exerted the most pronounced influence on the absolute signal intensities of the three virus detections, all exhibiting relatively high variation (as indicated by the R values).

The ki values, on the other hand, guides the determination of optimal factor levels, and the combination of these optimal levels is considered the most favorable loading buffer composition. According to the ki values, the optimal combination of factors and levels was found to be A2B2C2D1E3F1G2. Consequently, the final selected composition of the loading buffer included a 0.075M pH 8.5 Tris buffering system, containing 3 wt% sucrose, 1 wt% trehalose, 2 vt% Tween-20, 0.25 wt% PEG-20000, and 0.5 wt% BSA.

**Table S4** Orthogonal factor levels

| **Factor** | | **Levels** | | |
| --- | --- | --- | --- | --- |
|  |  | **1** | **2** | **3** |
| A | pH value | 7.5 | 8.5 | 9.5 |
| B | Ion concentration (mol/L) | 0.05 | 0.075 | 0.1 |
| C | Sucrose (wt%) | 1 | 3 | 5 |
| D | Trehalose (wt%) | 1 | 3 | 5 |
| E | Tween-20 (vt%) | 0.5 | 1 | 2 |
| F | PEG-20000 (wt%) | 0.25 | 0.5 | 1 |
| G | BSA (wt%) | 0.25 | 0.5 | 1 |

**Table S5** L18 (3^7^) orthogonal design and test results

| **NO.** | **A** | **B** | **C** | **D** | **E** | **F** | **G** | **Results** |
| --- | --- | --- | --- | --- | --- | --- | --- | --- |
| **1** | 1 | 1 | 1 | 1 | 1 | 1 | 1 | 18208 |
| **2** | 1 | 1 | 2 | 3 | 2 | 3 | 3 | 17297 |
| **3** | 1 | 2 | 1 | 2 | 2 | 3 | 2 | 18195 |
| **4** | 1 | 2 | 3 | 1 | 3 | 2 | 3 | 19043 |
| **5** | 1 | 3 | 2 | 2 | 1 | 2 | 1 | 19007 |
| **6** | 1 | 3 | 3 | 3 | 3 | 1 | 2 | 19517 |
| **7** | 2 | 1 | 1 | 2 | 3 | 2 | 3 | 20333 |
| **8** | 2 | 1 | 3 | 1 | 1 | 3 | 2 | 18113 |
| **9** | 2 | 2 | 2 | 3 | 1 | 2 | 2 | 19339 |
| **10** | 2 | 2 | 3 | 2 | 2 | 1 | 1 | 20548 |
| **11** | 2 | 3 | 1 | 3 | 3 | 3 | 1 | 18116 |
| **12** | 2 | 3 | 2 | 1 | 2 | 1 | 3 | 20368 |
| **13** | 3 | 1 | 2 | 2 | 3 | 1 | 2 | 18864 |
| **14** | 3 | 1 | 3 | 3 | 2 | 2 | 1 | 17772 |
| **15** | 3 | 2 | 1 | 3 | 1 | 1 | 3 | 18224 |
| **16** | 3 | 2 | 2 | 1 | 3 | 3 | 1 | 19828 |
| **17** | 3 | 3 | 1 | 1 | 2 | 2 | 2 | 19631 |
| **18** | 3 | 3 | 3 | 2 | 1 | 3 | 3 | 17368 |
| **k_1_** | 18545 | 18431 | 18785 | 19199 | 18377 | 19288 | 18913 |  |
| **k_2_** | 19470 | 19196 | 19117 | 19053 | 18969 | 19188 | 18943 |  |
| **k_3_** | 18615 | 19001 | 18727 | 18378 | 19284 | 18153 | 18772 |  |
| **R** | 925 | 765 | 390 | 821 | 907 | 1135 | 171 |  |
| **Influence order** | F>A>E>D>B>C>G | | | | | | | |
| **Optimal level** | A2 | B2 | C2 | D1 | E3 | F1 | G2 |  |
| **Optimal combination** | A2B2C2D1E3F1G2 | | | | | | | |

**Section S2.2 Optimization of the LFA strip**

**Fig. S3a** demonstrates the superior detection performance of the CN95 membrane when compared to other NC membranes in the QBs-based LFA Biosensor, particularly for the multiplex detection of three respiratory viruses. In **Fig. S3b**, it is evident that separately coating NC membranes with 0.5 mg/mL of SARS-CoV-2 antibodies, 0.8 mg/mL of ADV antibodies, and 0.8 mg/mL of IAV antibodies resulted in achieving the highest signal-to-noise ratio (SNR) for the detection of their corresponding target viruses. By analyzing the time required to reach saturation signal intensities, as depicted in **Fig. S3c**, we have determined that the optimal incubation time for the sample was 5 minutes. Furthermore, the optimal testing time for the LFA strip was assessed, as shown in **Fig. S3d**, revealing that 15 minutes of chromatographic time was suitable for efficient virus detection.


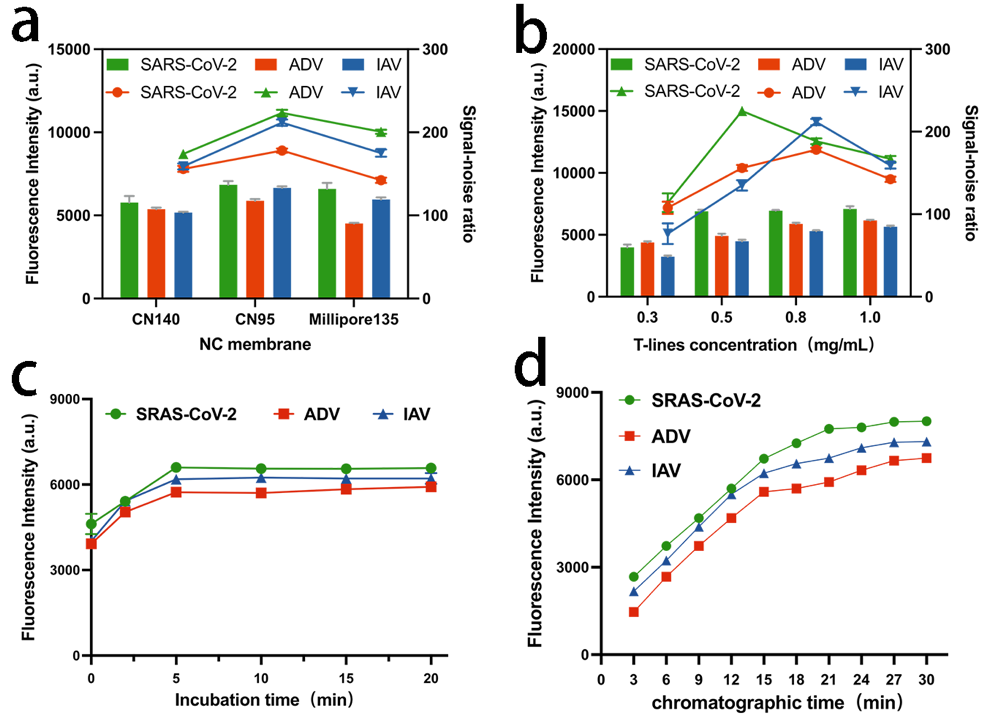


**Fig. S3.** Optimization of (a) NC membrane for the LFA biosensor test strip, (b) concentration of detection antibodies (anti-SARS-CoV-2, anti-ADV and anti-IAV) on the T-lines, (c) incubation time for samples and (d) reaction time of the LFA biosensor.

**Section S3 Evaluation**

**Section S3.1 Detection of SARS-CoV-2 NP antigen using the LFA biosensor.**

To assess the detection performance of our LFA Biosensor for the SARS-CoV-2 NP antigen, we conducted tests using varying concentrations of NP antigen with the proposed assay. **Fig. S4a** and **b** present both the images and corresponding fluorescence intensities of the strips designed for the NP antigen detection. We also constructed a calibration curve (**Fig. S4c**), which displayed a robust correlation with an R^2^ value of 0.9962. Importantly, the lowest detectable viral concentrations visible to the naked eye were as low as 0.06 ng/mL. Moreover, when determining the limit of detection (LOD) for the SARS-CoV-2 NP antigen testing, we followed the IUPAC protocol. The calculated LOD was approximately 5 pg/mL, showcasing the exceptional sensitivity of our assay.


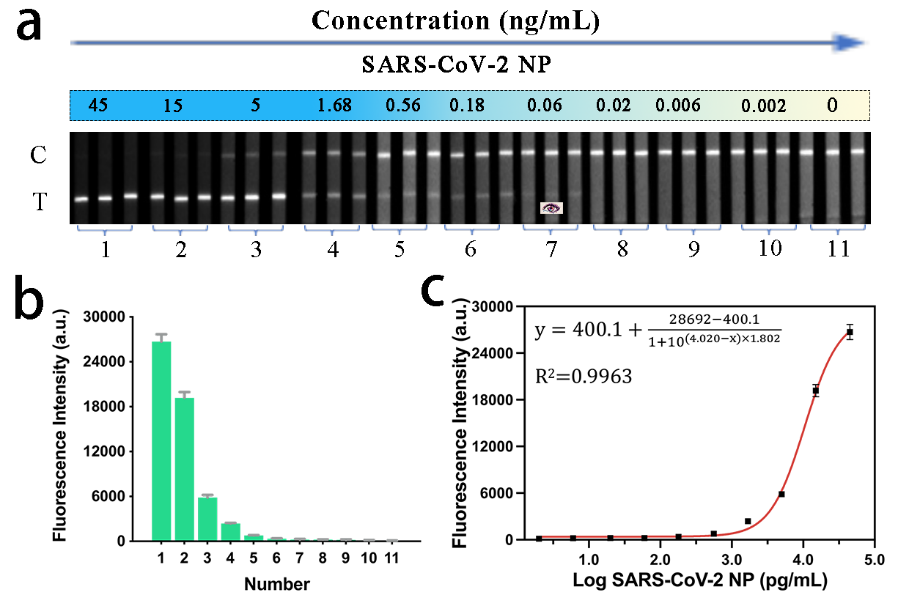


**Fig. S4.** (a) Images and (b) corresponding fluorescence intensities on T1 line of LFA strips for the detection of SARS-CoV-2 NP antigen. (c) Calibration curve for the detection of SARS-CoV-2 NP antigen.

**Section S3.2 LODs for three respiratory viruses determined through the RT-qPCR method**

Three viruses were diluted, and the concentrations of SARS-CoV-2 were diluted to 4.0 × 10^5^, 4.0 × 10^4^, 4.0 × 10^3^, 400, 40, and 4 copies/mL. The concentrations of ADV were diluted to 8.0 × 10^5^, 8.0 × 10^4^, 8.0 × 10^3^, 800, 80, and 8 copies/mL. The dilution concentrations of IAV were 3.0 × 10^5^, 3.0 × 10^4^, 3.0 × 10^3^, 300, 30, and 3 copies/mL. Using an ABI-7500 RT-qPCR instrument, the reaction condition is as follow: 50°C for 20 minutes, 95°C for 3 minutes, 95°C for 15 seconds, 55°C for 30 seconds, for 45 cycles. The virus amplification curves at different concentrations are shown in **Fig. S5**. The experimental results indicate that when the virus concentration of SARS-CoV-2, ADV, and IAV was 4, 8, and 3 copies/mL, respectively, positive results could not be found. It can be considered that Limit of Detection (LOD) estimation of the RT-qPCR method for the three respiratory viruses was 40, 80, and 30 copies/mL.


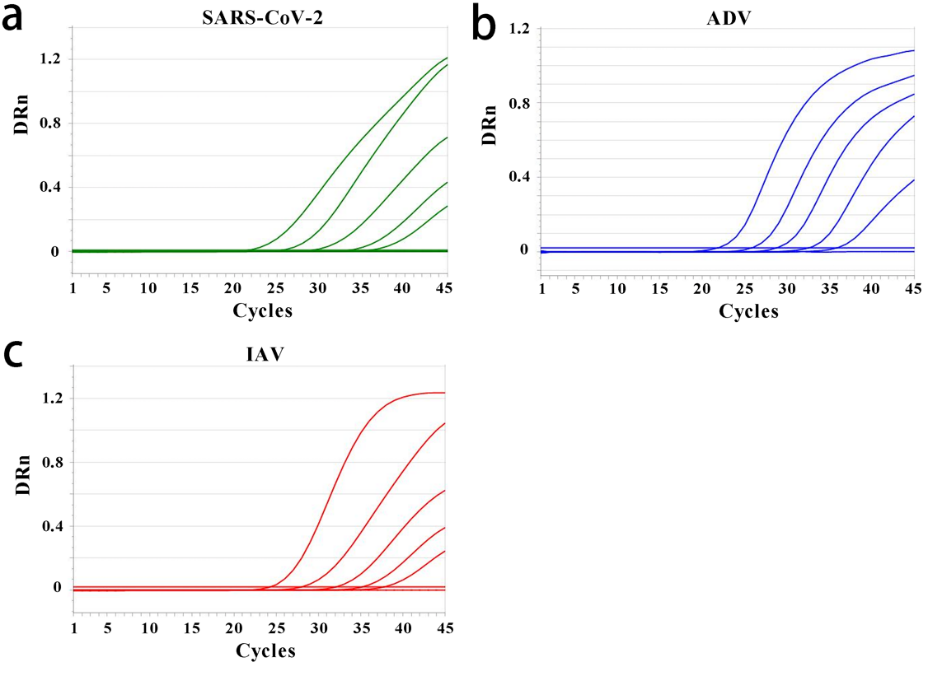


**Fig. S5.** Amplification curves of various concentrations of (a) SARS-CoV-2, (b) ADV, and (c) IAV by RT-qPCR.
